# Effectiveness of Silver Diamine Fluoride in Arresting Caries in Primary and Early Mixed Dentition: A Systematic Review

**DOI:** 10.3390/children9091289

**Published:** 2022-08-26

**Authors:** Zain Hafiz, Rehab Allam, Bdoor Almazyad, Alya’a Bedaiwi, Areej Alotaibi, Alwateen Almubrad

**Affiliations:** 1 Pediatric Dentistry and Orthodontics Department, College of Dentistry, King Saud University, Riyadh 12372, Saudi Arabia; 2 Intern, College of Dentistry, King Saud University, Riyadh 12372, Saudi Arabia

**Keywords:** children, caries arrest, primary teeth, first permanent molars, SDF

## Abstract

Dental caries is a painful condition that could lead to nutritional problems which affects the overall health of the child, as well it is expensive to treat. The effectiveness of silver diamine fluoride (SDF) in primary and early mixed dentition is systematically reviewed in this study. This systematic review utilized the Preferred Reporting Items for Systematic reviews and Meta- Analysis statement (PRISMA, 2020). A literature search conducted using the common electronic databases (from 2010–2021). Based on the inclusion and exclusion criteria, ten randomized clinical trials (RCT) have met the inclusion criteria and were considered for the qualitative assessment. Critical appraisal of these studies was done. This systematic review found that using SDF is a successful and effective method in arresting dentin carious process in the primary teeth and first permanent molars in children. Additionally, when compared to the commonly used topical fluoride products and materials, SDF showed to have better cariostatic effect. However, these findings must be cautiously viewed since more research is required to support them.

## 1. Introduction

Dental caries is a painful condition that could lead to nutritional problems which effect the general health of the child, as well it is costly to treat [1]. Early childhood caries has been presented by the World Health Organization (WHO) as a worldwide condition. It was reported that the prevalence of this disease is between 60% and 90% [2]. Moreover, the statistics issued by the European countries have showed that 61% of children (6–12) years old have at least one tooth affected with dental caries. As well, due to the widespread of dental caries, this disease may cause a financial load on the society besides its deteriorating results on the children’s oral health [3]. In addition, the prevalence of dental caries and its severity in Saudi children was found to be almost 80% for the primary teeth with a mean dmft of 5.0 and almost 70% for the permanent teeth with a mean DMFT score of 3.5 [4].

Conservative management is the modern approach in managing dental caries. It includes: the early recognition of non-cavitated lesions, identifying the child’s caries risk, identifying the activity of the disease, valid and reliable surveillance to select the appropriate conservative approaches and monitoring the signs of caries arrest or the progression of the carious lesions [5]. A systematic review by Gao et al. (2016) reported that the caries activity can be arrested by the application of topical fluoride without operative interventions [6].

Topically applied fluoride products, such as sodium fluoride (NaF) varnishes, are used for prevention due to their remineralization ability and their antimicrobial properties [7]. In 2014, the FDA has approved the use of sodium diamine fluoride (SDF) as a treatment for sensitive teeth, with an off-label use in arresting the carious process. However, it was recently approved (code D1354) to be used as an interim caries arrest product [8].

In addition to its cariostatic activity, 38% SDF causes an unfavorable environment for the activation of the dentin collagen enzyme [9]. Several studies have reported the effectiveness of SDF and NaF in arresting the caries process in primary and mixed dentition [10,11,12,13]. Chu et al. (2002) have studied the effectiveness of SDF and NaF in arresting dentin caries in 375 children (3–5) years old. The children were assigned into 5 groups. Children in the first and second groups have received a yearly administration of silver diamine fluoride solution (44,800 ppm F) after the soft carious dentin lesions have been excavated. Children in the third and fourth groups have received sodium fluoride varnish (22,600 ppm F) application every three months. Whereas the fifth group haven’t received any treatment and was considered as the control group. All the 5 groups have been followed up for 30 months. It has been found that the children in the control group have developed more new lesions than the children in other groups who have received treatment using SDF and NaF varnish [10].

Furthermore, the systematic review by Trieu et al. (2019) regarding the abilities of SDF and NaF varnish in arresting the progression of dentin caries concluded that the effectiveness of SDF in arresting the caries progression manifested to be twice the effectiveness of NaF at 30 months period. Thus, SDF is more effective than NaF in controlling the progression of dentin caries [11].

Furthermore, in the current literature, studies have showed the effectiveness of different types of topical fluoride products and materials in arresting caries including SDF, NaF, silver nitrate (AgNO_3_), novel nano-silver fluoride (NSSF) and glass ionomer cement (GIC) restorative material. However, the current available findings propose that SDF could be a successful product in arresting dental caries and an appropriate substitute of the widely used topical fluoride products and materials. This, however, needs a comprehensive and systematic evaluation of evidence into the clinical efficacy of each product or material in arresting caries in primary and early mixed dentition. In addition, although studies have shown that the SDF is effective in arresting dental carious lesions progression, the exact mechanism is not clearly stated. Articles that investigated the effectiveness of the SDF in arresting carious lesions have used different detection and evaluation methods as for observation, delivery techniques, aims, recruited populations, and conclusions. Therefore, the aim of this study is to review systematically the effectiveness of SDF in arresting carious lesions in primary and early mixed dentition.

## 2. Methods

This systematic review utilized the Preferred Reporting Items for Systematic reviews and Meta-Analysis statement (PRISMA, 2020).

### 2.1. Search Strategy

**Figure 1:**
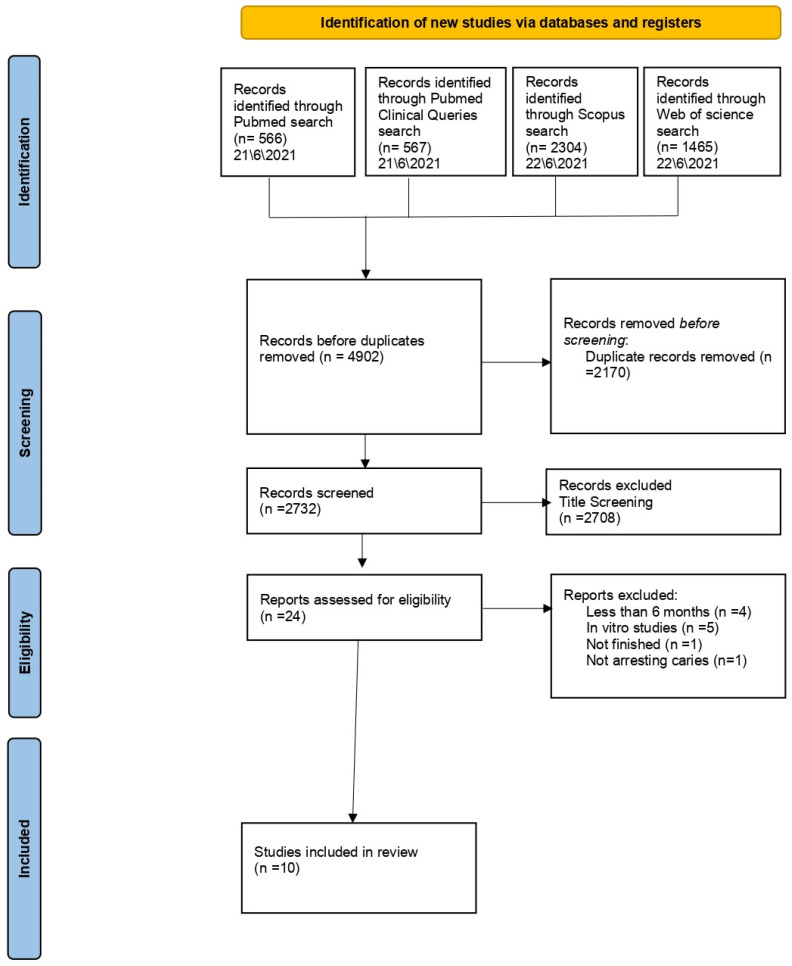
Flow diagram of the study selection process.

Searching the literature was done using the known electronic databases such as (PubMed, PubMed Clinical Queries, EMBASE, SCOPUS, Web of Science and Google Scholar) for studies that were published from 2010–2021. Key words that have been used in the search were: (fluoride) AND (arresting OR caries arrest OR non-active carious lesions) AND (caries in children OR early childhood caries). Moreover, the studies from 2010 to 2021 that contained the key words have been selected to formulate a potentially eligible list as the first screening process in this review (Figure 1).

### 2.2. Selection of Studies

Screening of the title and abstract has been done for the studies in the potentially eligible list that have been searched manually. The inclusion criteria were as follows: (1) randomized clinical trials (RCTs) (2) follow-up of at least six months (3) participants should be children who have primary dentition and/or first permanent molars (4) studies performed in humans (In vivo) (5) intervention: topical SDF solution, fluoride varnish (any concentration or frequency) and fluoride releasing restorative material (6) articles published in English. The exclusion criteria were as follows: (1) In vitro studies (2) clinical cases about home-use topical fluoride products (3) the follow-up was shorter than six months (4) study designs other than RCT (5) articles published before 2010.

Eventually, studies that have been selected, met the inclusion criteria. Full texts of the remaining studies were acquired. Studies that have been included in the assessment have been checked for their relevancy by manual search of their bibliographies. In addition, after the screening process, discussion of the selected articles was done by the co-investigators. If there were questions regarding an article and before reaching a decision, the article was discussed with the principal investigator (ZH). The percentages of arrested caries were calculated. As well, the risk of bias for each study was individually evaluated by the co-investigators (BM, AB, AO, AM). Finally, the results were discussed with the principal investigator (ZH).

### 2.3. Data Collection and Analysis

Prior to statistical analysis, data from the included articles were divided into two groups: caries arrest in primary dentition and caries arrest in first permanent molars. Using the original data that have been presented by the researchers of previous studies who investigated caries arrest in primary dentition, the numbers of carious lesions prior to and post management were obtained. As well, the percentages of controlled carious lesions for each study were obtained.

Moreover, to calculate the caries arrest rates in studies investigating caries arrest in first permanent molars; the baseline final numbers of carious lesions and the after intervention final numbers of arrested carious lesions were used. Based on the methods used by the investigators of the included studies to diagnose dental caries, carious lesions were recorded as active if they were soft upon light probing and arrested when they were hard. Finally, the method suggested by the Cochrane Handbook for Systematic Review of Interventions (the recommended tool is the revised version of the Cochrane tool, known as RoB 2). The RoB 2 tool provides a framework for assessing the risk of bias in a single result (an estimate of the effect of an experimental intervention compared with a comparator intervention on a particular outcome from any type of randomized trial) was implemented for the risk of bias for each study [14].

## 3. Results

### 3.1. Study Selection

Publications were systemically searched by four investigators using six online databases: PubMed, PubMed Clinical Queries, EMBASE, SCOPUS, Web of Science, and Google Scholar. The keywords used were (fluoride) AND (arresting OR caries arrest OR non-active carious lesions) AND (caries in children OR early childhood caries). The years of when the articles have been published were selected from 2010 to 2021. A total of 174 articles were identified, after the removal of duplicated and initial screening, 40 papers were evaluated for eligibility criteria after the full text was independently reviewed. Finally, 10 articles met the inclusion and exclusion criteria.

### 3.2. Study Characteristics

Ten RCTs met the inclusion criteria which were published between 2010 and 2021. One study was conducted in India [15], one in Thailand [16], five articles were conducted in China [12,13,17,18,19], two in Brazil [20,21], and one in the Philippines [22]. A total of 4475 children with a mean age of two years old [16], three years old [20], three and a half years old [12,13,17,18,19], seven years old [22], and eight years old [15] were recruited in the included studies. Dropout rates were recorded in all articles except for one [21]. But all the included studies have done sample size calculations and collected the demographic background. Two studies focused on carious lesions in posterior teeth only [14,22]. While other RCT studied both posterior and anterior teeth. Three RCT used 30% SDF [12,17,20,21]. While the other studies used 38% SDF. One article used two different percentages of SDF 12% and 38% [19]. Five articles compared the effectiveness of SDF to NaF varnish and the rest of the articles compared it to other topical fluoride products and materials [18,19,20,21,22].

Children were clinically examined using dental probes and dmfs (decayed, missing, and filled surfaces) index for recording the outcomes by visual inspection and tactile detection of carious lesions in all the included RCT. Out of all the included RCT only three did not evaluate the oral hygiene at home by parental questionnaire, instead they did a follow up visit [14,21,22]. Moreover, the included studies used Cohen’s kappa statistics for assessing the intra-examiner reliability.

### 3.3. Study Findings and Outcomes

Monse et al. (2012) assigned children randomly from eight schools for 38% SDF application or atraumatic restorative treatment (ART) sealant. Two out of the eight schools’ children didn’t receive any treatment and were considered the control. The children were divided into brushing and non-brushing groups. After 18 months, it was found that the caries incidence in the SDF treated group didn’t significantly differ from the non-treated group. Furthermore, children who received the 38% SDF treatment and sealant had lower caries incidence. In the toothbrushing group, both the SDF group and non-treatment group were comparable to each other [22]. Moreover, Zhi et al. (2012) conducted a study comparing the effectiveness in arresting dentin caries by annual application of 38% SDF, semi-annual application and annual application of GIC. After 24 months the group that received application of 38% SDF every six months had higher caries arrest rate than the other two groups [18].

Dos Santos et al. (2012) found that the effectiveness of 38% SDF in arresting caries is more than the interim restorative treatment (IRT) using GIC fillings with statistically significant difference (*p* < 0.05) [21]. Furthermore, Duangthip et al. (2016–2018) established three groups to compare the application of 30% SDF and 5% NaF varnish (annual application of 30% SDF, three applications of 30% SDF at weekly intervals, and three applications of 5% NaF at weekly intervals) in arresting caries activity in primary dentition with follow-up examinations semi-annually. Thirty months later, the annual applications of 30% SDF solution group had arrested carious lesions which were significantly higher than the group who received three applications of 30% SDF and 5% NaF varnish at weekly intervals (*p* < 0.001) [12,17].

Fung et al. (2018) compared the effectiveness of two different concentrations of SDF (12% and 38%) on arresting the caries activity and found at the 30 months follow up that the 38% SDF group had higher caries arrest rate than the 12% SDF [19]. In addition, Tirupathi et al. (2019) have randomly divided 50 children into two groups: 38% SDF group and 5% NSSF group to compare their cariostatic efficacy. After 12 months follow up, there was no statistically significant difference between the arrested lesions and the number of active carious lesions in the 38% SDF group and 5% NSSF group (*p* > 0.05). Therefore, the annual application of 38% SDF has the same efficacy in arresting dentinal caries as 5% NSSF [15].

Vollú et al. (2019) also compared the efficacy of 30% SDF to ART on carious lesions. At the 12 months follow up, it was found that the mean difference of controlled lesions between the groups was −0,07(0.05; −0.17–0.30) and the application of SDF was more time-efficient (shorter time) than ART (*p* < 0.001) [20]. Additionally, Gao et al. (2020) compared two groups (Group A: received 25% AgNO_3_ solution + 5% NaF varnish, Group B: received 38% SDF solution + placebo varnish) and found that application of 38% SDF every six months is as effective as the application of 25% AgNO_3_ every six months followed by 5% NaF in controlling early childhood caries (ECC) [13].

Mabangkhru et al. (2020) conducted a randomized clinical trial on children 1–3 years of age to compare the efficacy of the application of 38% SDF and 5% NaF varnish on arresting caries. The multilevel logistic regression analysis showed that the 38% SDF application was more effective in arresting caries than 5% NaF varnish application [16].

Table 1 shows the details of all included studies.

### 3.4. Quality Assessment

**Figure 2:**
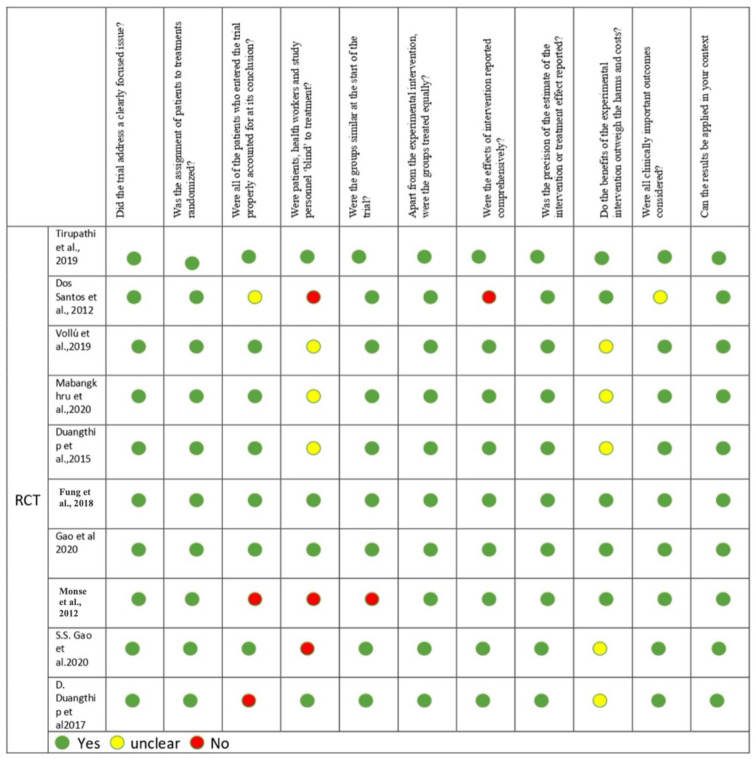
Quality assessment of the included studies by CASP tool [6,12,13,15,16,17,19,20,21,22].

The quality of the articles was evaluated using the CASP tool (Figure 2). Ten studies have showed a high quality of evidence as they fulfilled scores of 11 to 8. A study by dos Santos et al. (2012) was evaluated to provide a moderate level of evidence with a score of 7 [21]. In all the included studies, patients were assigned into groups by stratified block randomization, while one study by Monse et al. (2012) didn’t use any randomization [22]. The assessment of intra-examiner reliability was done using Cohen’s Kappa statistics. Moreover, three RCTs utilized double blinding protocol [12,13,19]. The authors dos Santos et al. (2012) and Vollú et al. (2019) were unable to conduct a double-blind or single-blind study due to the differences in the materials [20,21]. Three of the articles didn’t mention any blinding protocol [12,16,22]. Clinically significant outcomes were obtained, and all the articles mentioned the dropout rates except in dos Santos et al. (2012) in which they didn’t mention the final dropout rate [21].

## 4. Discussion

Dental caries is a global chronic bacterial disease that demineralizes the hard tissues of the dentition and remains a significant issue mostly in populations with low socioeconomic conditions [20]. Preventive management of dental caries should be taken in every community such as fluoridation of public water, using dentifrices with fluoride, good oral hygiene implementation and induce healthy dietary habits through the available community educational methods [15]. This systematic review showed that SDF is more effective than NaF in arresting caries in both primary and permanent teeth in school age children. After reviewing 40 papers for the eligibility criteria, 10 articles met the inclusion criteria for this systematic review. The quality of each study was examined based on the CASP protocols for RCTs. All the articles have mentioned the dropout rates except in dos Santos et al. (2012) study which may cause an attrition bias [21]. Moreover, almost all the studies have high evidence level that agree with their outcomes since they have met the CASP criteria. Zhi et al. (2012) and dos Santos et al. (2012) found that SDF is more effective in arresting caries than GIC used in ART when applied bi-annually which emphasizes on the importance of the follow up visits to achieve the desired cariostatic effect [18,21]. In contrast, Monse et al. (2012) and Vollú et al. (2019) compared SDF and ART effect on carious lesions and found no significant difference in their cariostatic efficacy. However, the SDF had less chair-time than ART [20,22].

Furthermore, the results of Duangthip et al. (2016) and its follow up article (2018) compared 30% SDF and 5% NaF varnish in controlling caries and found that 30% SDF is more effective than 5% NaF varnish [12,17]. In agreement, Mabangkhru et al. (2020) compared the effect of 38% SDF and 5% NaF varnish in arresting caries and concluded that the application of 38% SDF is more effective than 5% NaF varnish [16]. Moreover, Fung et al. (2018) who compared two different SDF concentrations reported that 38% SDF is more effective in arresting caries than 12% SDF, indicating that the SDF concentration has a significant role in the caries arrest process [19]. The results of Tirupathi et al. (2019) showed that using 38% SDF and 5% NSSF have the same efficacy in arresting caries which was explained by the synergism of NSSF components (Nano silver and sodium fluoride) [15]. In contrary, Gao et al. (2020) found the use of 38% SDF every six months was as effective as the use of 25% AgNO_3_ followed by the application of 5% NaF every six months in arresting ECC, and this can be explained by the high fluoride concentration in SDF (44,880 ppm) [13]. In addition,, the limitations mentioned in the studies such as small sample size and conducting the studies in one site may not be representative of the population [14,18,21]. Additionally, because radiography was infeasible in the community, Mabangkhru et al. (2020) used the visual-tactile examination for detecting caries which was considered as a limitation in their study. As well, it was mentioned that the black staining of SDF may lead to a bias on detecting caries. More limitations were mentioned such as using a blinded trained examiner who was not involved in the treatment and the 12-month study period that was considered a short period for confirming the caries progression and activity [16].

Gao et al. (2020) mentioned that the limitations of their study included the follow up period (six months interval) between the examinations as they were not able to determine the time when the caries process was arrested. Also, the reported caries arrest effectiveness might be lower than the real number since they used LOCF (last observation carried forward method) to input missing data. In addition, conducting the trial in a place with compromised equipment was one of the limitations because the caries arrest rate would be higher if the study was conducted in clinical setting [13]. Thus, this systematic review has to be cautiously viewed since it encountered limitating factors such as the different caries detection techniques used in the studies and the difficulty to blind the operator and examiner from the treatment groups since the SDF has different texture and causes black staining of teeth unlike NaF and the other topical fluoride products and materials [13,16]. This may play a role in representing the results of the studies.

In summary, SDF shows more effectiveness in arresting caries than the available topical fluoride products and materials in primary and permanent dentitions. The application of SDF is simple, less time consuming, and doesn’t require high operator’s skill. It is considered inexpensive compared to other materials such as GIC, also it is easy applied in teeth with difficult accessibility. However, the main disadvantage of using SDF is the dark black staining color.

Many studies have reported different solutions to overcome this disadvantage and decreasing the staining of the treated teeth such as the application of potassium iodide following the application of SDF [23,24,25,26]. In terms of parental acceptance, several studies have reported that SDF black staining of teeth was accepted by parents in posterior compared to anterior teeth in addition to other reasons as the child’s cooperation, socioeconomic status, and the need of using sedation or referral to general anesthesia to receive dental treatment [12,16,20].

Finally, the quality of evidence of this systematic review was based on 10 articles which reported that using 38% SDF will lead to caries arrest in both primary and permanent dentitions. However, additional research is needed to specify the frequency of application and the optimum follow up intervals for its cariostatic efficacy.

## 5. Conclusions

Based on the findings of this systematic review, the application of SDF is a practical and efficacious practice in arresting dentin carious lesions in primary dentition and first permanent molars in children. As well, SFD showed to be more effective in arresting caries in primary and early mixed dentition when compared to the known used topical fluoride products and materials. Nevertheless, these findings must be cautiously viewed since more research is required to support them.

## Figures and Tables

**Table 1:** Data extraction sheet and characteristics of included articles.

Year	Article	Aim	Population	Design	Intervention	Comparison	Outcome	Drop-Out	Duration	Location	Statistical Analysis	Results
2012	Monse et al. [22]	Comparing the effectiveness of single application of 38% SDF * to ART ** sealants in arresting caries	1016 Children 6–8 years	RCT	Group 1 SDF application brushing Group 2 SDF application non-brushing Group 3 ART sealant brushing Group 4 ART sealant non-brushing	Group 5 Non-treated brushing Group 6 Non-treated non-brushing	- Caries was scored according to WHO - Intact surfaces - Number of surfaces with new (D3) carious lesions - Caries incidence	312 children	18 months	Philippines	SAS 9.1 software.Kappa chi-square tests	The non-treatment Group had Higher percent. of D3 occ. Surfaces
2012	dos Santos et al. [21]	Comparing 30% SDF and IRT *** in arresting caries in school children	91 males and females 5–6 years	RCT	30% SDF group - No caries excavation, cotton rolls were used to isolate the teeth from saliva, then application of SDF for 3 months - Evaluations at 6 and 12 months.	IRT group - No caries excavation, cotton rolls were. cavity conditioner before sealing the cavities with GIC - Evaluations at 6 and 12 months	- dmft - Caries arrest	Not mention	12 months	Brazil	SPSS Descriptive statistical Chi-squared test Fisher’s exact test Cohen’s kappa test	After 12 months, 30% SDF was more effective in arresting caries than IRT [relative risk (RR = 66.9%)]
2012	Zhi et al. [18]	Comparing the effectiveness of: - yearly application of SDF application of 38% SDF every six months - and yearly application of GIC **** - in arresting active dentin caries in primary teeth	212 males and females 3–4 years	RCT	Group 1 The superficial soft decayed tissues were removed by hand instruments, then application of 38% SDF every 12 months Group 2 same procedure as mentioned above except that the application of 38% SDF was every 6 months	Group 3 The superficial soft decayed tissues were removed by hand instruments, conditioner was usen then GIC was applied every 12 months	Arrest of active carious lesions in primary dentition	20%	24 months	China	SPSS 16.0 for Windows Chi-square test ANOVA Multilevel non-linear logistic regression model	After 24 months the group who received semi-annual application of 38% SDF (every 6 months) had higher caries arrest rate than the other two groups (OR = 2.98, *p* = 0.007)
2016	Duangthip et al. [17]	Comparing the effectiveness of three topical fluoride application methods: 1.Annual application of 30% SDF, 2.three applications of 30% SDF at weekly interval at baseline, 3.three applications of 5% NaF ^ varnish at weekly interval at baseline in arresting dentin caries in primary teeth of preschool children in a fluoridated area.	304 Children 3–4 years	RCT	Group 1 Annual application of 30% SDF	Group 2 Three applications of 30% SDF at weekly interval at baseline Group 3 Three applications of 5% NaF at weekly interval at baseline	- Presence of plaque - VPI §§ - dmfs index Status (inactive/active) of the carious lesions (ICDAS codes 5–6)	29 children	18 months	Guangzhou, China	SPSS 20.0 for Windows (SPSS Inc., Chicago, IL, USA) and WinBUGS 1.4. Cohen’s Kappa statistics. Chi- square test. ANOVA	Result of the multi-level survival analysis showed that the two SDF application methods could lessen the time to arrest dentin caries compared with the NaF application method
2018	Fung et al. [19]	Comparing the effectiveness of different concentrations of SDF solution (12% or 38%)	888 children 3–4 years	RCT	Group 1 Annual application of 12% SDF Group 2 Semi-annual application of 12% SDF	Group 3 Annual application of 38% SDF Group 4 Semi-annual application of 38% SDF	Caries arrested dmft/dmfs	89 children	30 months	Hong Kong, China	(SPSS, Inc.) Kappa ANOVA Chi-square tests McNemar test Generalized estimating equations (GEE) (QICC)	Using 38% SDF resulted in higher caries arrest than 12% SDF (odds ratio [OR], 1.98; 95% confidence interval [CI], 1.51–2.60, *p* < 0.001)
2018	Duangthip et al. [12]	Comparing the effectiveness of three methods in applying SDF: - annually - three applications of SDF solution or—NaF varnish at weekly interval at baseline in arresting active dentin caries	371 children 3–4 years	RCT	Group 1 30% SDF applied every 12 months Group 2 Three applications of 30% SDF at weekly intervals	Group 3 Three applications of 5% NaF at weekly intervals	- dmfs - Caries arrest - VPI - Extent of carious lesions (ICDAS codes 3 to 6) - Color of lesion (yellow, light brown, hard brown, or black) - Presence of plaque	60 children	30 months	Hong Kong, China	Cohen’s Kappa statistics SPSS 20.0 for Windows (SPSS Inc., Chicago, IL, USA) GLIMMIX procedure SAS/STAT^®^ software version 9.3 (SAS Institute Inc., Cary, NC, USA)	The effect of caries arrest when applying SDF every 12 months was found to be more than 3 weekly applications of NaF or SDF at baseline
2019	Vollú et al. [20]	Investigate the efficacy of SDF in arresting active caries in primary molars The followings were evaluated: - Time needed for treatment, - side effects, - aesthetic perception by parents - anxiety, - and oral health related to quality of life (OHRQoL)	68 Children 2–5 years	RCT	Group 1 SDF	Group 2 ART	- dmft/DMFT index - ICDAS index - Oral health related to quality of life (OHRQoL) and anxiety - Time needed for the treatment - Side effects - Parental aesthetic perception	15 children	12 months	Brazil	chi-square Fisher’s exact tests Shapiro Wilk test Mann Whitney test Student *t*-test Indicator of Positive Change (IpC) Cohen Mann Whitney test	-The time required to treat with SDF was lower than the ART (*p* < 0.001). -There was no difference in the percentage of side effects, aesthetic perception (*p* = 0.709), and the change in anxiety (*p* = 0.155). OHRQoL was less effected after ART, but only when the parents’ distress subscale was considered (*p* = 0.012).
2019	Tirupathi et al. [15]	Evaluate the effectiveness of of 5% NSSF ^^ dental varnish with 38% SDF in arresting dentinal caries in primary molars	50 children (both genders) Group A: mean age 7.88 ± 1.30 Group B: Mean age 8.39 ± 1.41	RCT	5% NSSF Group No caries excavation Application of single drop (0.1 mL) of 5% NSSF, no repetition	38% SDF Group No caries excavation. Application of single drop (0.1 mL) of 38% SDF, no repetition	Caries arrest ability of 5% NSSF to that of 38% SDF in preventing progression of carious lesions in primary molars Size of carious lesion - Depth of the lesion - Pain - Carious lesion activity Overall failure or success	3 children (7.5%)	12 months	Government primary school—India	SPSS for Windows release 19.0 Cohen kappa Chi-square test ANOVA 95% confidence interval tukey post-hoc test	There was no significant difference in the number of the arrested and active carious lesions in the 38% SDF group and 5% NSSF group (*p* > 0.05) at the 12-month follow up
2020	Mabangkhru et al. [16]	Comparing the cariostatic efficacy of 38% SDF solution, and 5% NaF varnish when applied every six months in young children with high caries risk	284 Children 1–3 years	RCT	Group 1 38% SDF	Group 2 5% NaF	- VPI - dmft index Carious lesion activity	21 children	12 months	Thailand	SPSS 20.0 for Windows Cohen’s kappa statistics chi-square test *t*-test or Mann–Whitney U test	-Mean dmfs scores in Groups 1 and 2 were 8.89 and 9.79, respectively. The results of the multilevel logistic regression analysis showed that the intervention in Group 1 was more effective in arresting carious lesions than that of Group 2 (OR = 2.04; 95% CI, 1.41–2.96).
2020	Gao et al. [13]	Comparing the effectiveness of 25% AgNO_3_ ^^^ + 5% NaF and 38% SDF in arresting ECC §	1070 children 3 years	RCT	Group 1 25% AgNO_3_ solution + 5% NaF varnish	Group 2 38% SDF solution + placebo varnish	- dmfs - Caries arrest - Active ds - VPI	190 children	30 months	Hong Kong, China	G*Power version 3.1.7, Kiel, Germany -Statistical Package for the Social Sciences 24.0 (SPSS Inc., Chicago, IL, USA).	The mean number of inactive ds in Groups A and B were 3.65 ± 3.62 and 3.56 ± 3.69, respectively (*p* = 0.694) at the 30-month follow up

* SDF: Sodium Diamine Fluoride. ** ART: Atraumatic Restorative Treatment. *** IRT: Interim Restorative Treatment. **** GIC: Glass Ionomer Cement. ^ NaF: Sodium Fluoride. ^^ NSSF: Novel Nano-Silver Fluoride. ^^^ AgNO_3_: Silver Nitrate. § ECC: Early Childhood Caries. §§ VPI: Visible Plaque Index.

## Data Availability

Data supporting the findings of the present study can be requested from authors.
